# Deletion of Viral microRNAs in the Oncogenesis of Epstein–Barr Virus-Associated Lymphoma

**DOI:** 10.3389/fmicb.2021.667968

**Published:** 2021-07-08

**Authors:** Hiroshi Kimura, Yusuke Okuno, Yoshitaka Sato, Takahiro Watanabe, Takayuki Murata

**Affiliations:** ^1^Department of Virology, Nagoya University Graduate School of Medicine, Nagoya, Japan; ^2^Medical Genomics Center, Nagoya University Hospital, Nagoya, Japan; ^3^PRESTO, Japan Science and Technology Agency, Kawaguchi, Japan; ^4^Department of Virology and Parasitology, Fujita Health University School of Medicine, Toyoake, Japan

**Keywords:** EBV, microRNA, BART, lymphomagenesis, CAEBV, ENKTL, diffuse large B cell lymphoma

## Abstract

Epstein–Barr virus (EBV), which encodes >80 genes and nearly 50 non-coding RNAs, is a double-stranded DNA virus. EBV is associated with various types of lymphomas and lymphoproliferative disorders not only of B-cell but also T/NK-cell origin. However, the oncogenic mechanism remains poorly understood, including the EBV receptors expressed on T/NK cells, relationship of EBV with host genes, and epigenetic regulation of EBV and host genes. The roles of host and viral non-coding RNAs during tumorigenesis have been elucidated. EBV encodes at least 49 mature microRNAs (miRNAs), of which 44 are located in *Bam*HI-A rightward transcripts (BARTs) region, and the remaining five are located in *Bam*HI-H rightward fragment 1. BART miRNAs modulate cell differentiation, proliferation, apoptosis, and the cell cycle, and they are considered positive regulators of oncogenesis. We and others have recently reported that EBV-positive lymphomas frequently possess large deletions in BART miRNA clusters, suggesting that some viral miRNAs have suppressive effects on oncogenesis, and that deletion of these miRNAs may aid lymphoma formation.

## Introduction

Epstein–Barr virus (EBV), which is the first human oncovirus, was isolated from Burkitt lymphoma by Epstein et al. (1964). EBV is a 170–180 kb double-stranded DNA virus belonging to the herpesvirus family and gammaherpesvirus subfamily, and its genome encodes approximately 80 genes (Longnecker et al., 2013). EBV belongs to the same subfamily as Kaposi’s sarcoma-associated herpesvirus, which is the causative virus of Kaposi’s sarcoma, and both viruses infect B cells and are related to B-cell lymphomas. In addition to Burkitt lymphoma, EBV is associated with a variety of malignancies with B-cell origins, such as Hodgkin lymphoma, diffuse large B-cell lymphoma (DLBCL), and immunodeficiency-related lymphoproliferative disorders detected after organ/hematopoietic stem cell transplantation (Swerdlow et al., 2016).

Epstein–Barr virus is also associated with natural killer (NK)-cell and T-cell neoplastic diseases including extranodal NK/T-cell lymphoma, nasal type (ENKTL) (Chan et al., 2017), chronic active EBV disease (CAEBV) (Quintanilla-Martinez et al., 2017; Cohen et al., 2020), and epithelial tumors such as nasopharyngeal carcinoma and gastric cancer (Cohen, 2000; Longnecker et al., 2013). Although extensive studies have been conducted on the implication of MYC translocation and activation in Burkitt lymphoma (Longnecker et al., 2013), the oncogenic mechanisms (including the T/NK-cell receptors expressed, the relationship of EBV with host genes, and epigenetic regulation of EBV and host genes) associated with other lymphoid tumors are still unclear.

The roles of host and viral non-coding RNAs have been elucidated during tumorigenesis in various malignancies, including EBV-associated diseases (Cai et al., 2006; Qiu et al., 2011; Klinke et al., 2014; Kuzembayeva et al., 2014; Dreyfus, 2017; Iizasa et al., 2020). We and others have recently reported that EBV-positive lymphomas frequently possess large deletions in viral microRNA (miRNA) clusters, suggesting that some miRNAs negatively regulate lymphomagenesis (Peng et al., 2018; Okuno et al., 2019; Mabuchi et al., 2021). This review outlines the role of viral non-coding RNAs in the lymphomagenesis of EBV-associated diseases, focusing on EBV-encoded miRNAs.

### Biology of EBV Infection

Epstein–Barr virus can cause either a latent or lytic infection. In a latent infection, EBV exists as an episome in the nucleus. No viral particles are produced in the host cell, and only a limited number of genes are expressed. Latent infections are classified into four patterns, type 0, I, II, and III, depending on the host cell, tissue, and immune status (Longnecker et al., 2013; Munz, 2019). Latency type 0 is seen in the memory B cells of healthy individuals, and few viral proteins are expressed in this type. In type I, EBV nuclear antigen (EBNA) 1 and EBV-encoded small RNAs (EBERs) are expressed. In a type II infection, latent membrane protein (LMP) 1 and LMP2 are additionally expressed. EBNA2, 3s, and LP are expressed in type III infections.

In contrast, immediate-early, early, and late genes are expressed one after another during lytic infection, and virus particles are produced. It was previously considered that virus-infected cells are immediately in a latent state after EBV infects B cells. However, recent evidence suggests that these cells are temporarily in an abortive lytic infection state, and that the expression of EBV lytic genes plays an important role in the immortalization of infected cells (Ma et al., 2011; Murata et al., 2014; Munz, 2019). In fact, these lytic genes are expressed in hydroa vacciniforme-like lymphoproliferative disorder and DLBCL (Yamamoto et al., 2016; Cohen et al., 2018), which suggests that the abortive lytic infection may be involved in lymphomagenesis.

### Oncogenesis of EBV and EBV-Related Malignancies

Epstein–Barr virus is used to immortalize human B cells *in vitro* to produce a lymphoblastoid cell line (LCL). LMP1 is a viral membrane oncoprotein essential for immortalization; it mimics CD40 expressed by T cells and constantly activates downstream NF-κB, PI3K/AKT, JNK, and p38/MAPK pathways, immortalizing infected cells and suppressing apoptosis (Kanda, 2018). Although LMP2A is not essential for immortalization, it is also an oncoprotein that mimics the B-cell receptor expressed by B cells, resulting in constitutive calcium recruitment, protein kinase C activation, cell proliferation, and differentiation suppression (Longnecker et al., 2013). EBNA1, EBNA2, EBNA3A, EBNA3C, and EBNA-LP are nuclear proteins that help transform B cells and maintain latency (Tomkinson et al., 1993; Kanda, 2018). All these latent infection-related genes are expressed in a type III infection where host cell-mediated immunity is suppressed, such as immunodeficiency-related lymphoproliferative disorders. As only a limited number of viral genes are expressed during latent type I and II infections, it is easier to avoid host immunity with these infections compared with a type III infection. However, immortalization and suppression of apoptosis by viral oncoproteins are limited in these latency types (Kimura, 2018).

Table 1 summarizes the latent infection patterns of EBV-related malignancies, as well as the degree of EBV involvement, type of cell infected, associated high-risk factors, and EBV miRNA profile. As mentioned above, EBV is associated with a variety of lymphomas and lymphoproliferative diseases, not only of B-cell but also T/NK-cell origin, as well as with epithelial malignancies. All malignancies with T/NK- or epithelial-cell origins are associated with type I and type II latency, in which a limited number of viral genes are expressed and evasion of host immunity is easier (Longnecker et al., 2013). Notably, some EBV-positive tumors have uneven distributions in specific areas as shown in Table 1; whether this is due to genetic variations in the host or differences in the infecting strains remains unclear.

**TABLE 1:** Representative EBV-associated malignancies and their characteristics.

Diseases	EBV association	Infected cells	Type of latency	EBV miRNA profile	High risk population
Burkitt lymphoma, endemic	>95%	B	I	miR-BART15-3p (Haneklaus et al., 2012) miR-BART16-5p (Hooykaas et al., 2017) miR-BART18-5p (Qiu and Thorley-Lawson, 2014)	Children in equatorial Africa, New Guinea
Hodgkin lymphoma, mixed cellularity	75%	B	II	miR-BART2-5p (van Eijndhoven et al., 2016) miR-BART13-3p (Sakamoto et al., 2017) miR-BART19-3p (Sakamoto et al., 2017)	
Lymphomatoid granulomatosis	100%	B	II		Westerners
EBV-positive diffuse large B cell lymphoma, not otherwise specified (DLBCL)	100%	B	II or III	miR-BHRF1-2-5p (Chen et al., 2019) miR-BHRF1-3-5p (Xia et al., 2008) mir-BART2 (Xia et al., 2008) mir-BART13 (Higuchi et al., 2018)	Individuals > 50 years
Post-transplant lymphoproliferative disorders	70%	B	III	All BHRF1 miRNAs and BART miRNAs (Fink et al., 2014)	Recipients with heart, lung, or intestine transplantation
Plasmablastic lymphoma	60–75%	Plasmablasts	I		HIV-infected individuals
Aggressive NK cell leukemia	>90%	NK	II		Asians
Extra nodal NK/T cell lymphoma, nasal type (ENKTL)	100%	NK, T	II	miR-BART8-3p (Huang and Lin, 2014) miR-BART8-5p (Huang and Lin, 2014) miR-BART20-5p (Huang and Lin, 2014)	East Asians
Systemic EBV-positive T cell lymphoma of childhood	100%	T	II		East Asians
Chronic active EBV disease of T/NK type (CAEBV)	100%	T, NK	II	miR-BART1-5p (Kawano et al., 2013) miR-BART2-5p (Kawano et al., 2013)	East Asians
Severe mosquito bite allergy	100%	NK, T	II		East Asians
Hydroa vacciniforme-like lymphoproliferative disorder	100%	γδT, NK	II		Asians, Native Americans
Nasopharyngeal carcinoma	100%	Epithelial	II		Adults in southern China and Southeast Asia
Gastric cancer	9%	Epithelial	I		

### EBV Non-coding RNAs

In addition to viral proteins, EBV encodes many non-coding RNAs that also potentiate oncogenesis. EBER1 and EBER2, which are long non-coding RNAs of 167 and 172 nucleotides, respectively, are expressed most abundantly in EBV latently infected cells at 10^7^ copies per cell (Arrand and Rymo, 1982; Howe and Shu, 1989). EBERs interact with a variety of RNA-binding proteins and optimize B-cell transformation (Yajima et al., 2005; Fok et al., 2006). However, the exact role of EBERs is still unknown (Munz, 2019). EBV also encodes at least 49 mature miRNAs. Of these 44 are located within the intronic regions of *Bam*HI-A rightward transcripts (BART miRNAs), and the remaining five are located in the *Bam*HI-H rightward fragment 1 (BHRF1 miRNAs) (Klinke et al., 2014; Iizasa et al., 2020).

*Bam*HI-H rightward fragment 1 miRNAs are expressed during EBV latency type III and lytic infections. These miRNAs inhibit apoptosis and promote cell cycle progression and proliferation to induce early-phase infection in B cells (Seto et al., 2010). In addition, BHRF1 miRNAs regulate time-restricted expression of the BHRF1 protein (viral BCL-2 homolog) to optimize B-cell transformation (Bernhardt et al., 2016).

**FIGURE 1:**
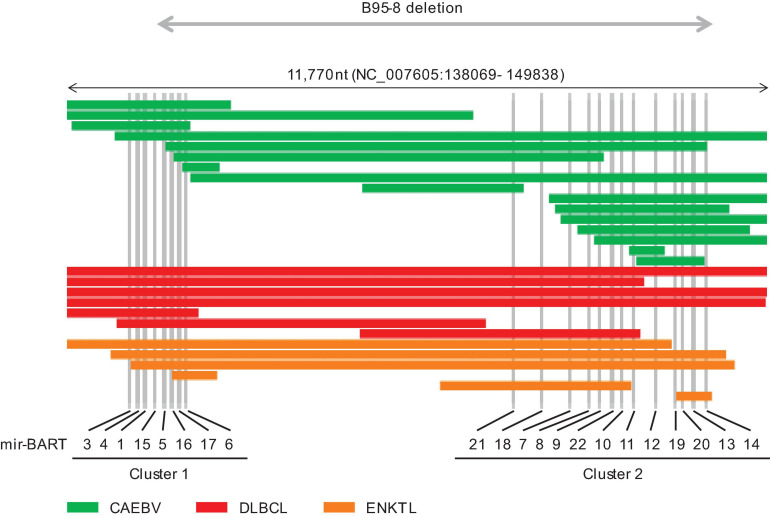
Distribution of intragenic deletions in *Bam*HI-A rightward transcript (BART) microRNA clusters 1 and 2. CAEBV; chronic active EBV disease of T/NK type, DLBCL; diffuse large B cell lymphoma, not otherwise specified, ENKTL; extranodal NK/T cell lymphoma, nasal type.

*Bam*HI-A rightward transcript miRNAs are expressed in latency types 0, I, II, and III (Amoroso et al., 2011). Their expression is regulated by both the immediate-early protein BZLF1 and feedback loops that involve the host transcription factor NF-κB (Dreyfus, 2017). BART miRNAs are mainly categorized into clusters 1 and 2 (Figure 1; Klinke et al., 2014), which encode eight and 13 pri-miRNAs, respectively. BART miRNAs target host genes and modulate cell differentiation, proliferation, apoptosis, and the cell cycle to establish infection and produce progeny viruses (Klinke et al., 2014). For, example, miR-BART1-3p and miR-BART16 target CASP3 and inhibit apoptosis (Vereide et al., 2014). BIM, a pro-apoptotic protein of the bcl2 family, is targeted by several BART miRNAs (Marquitz et al., 2011). Another BART miRNA (miR-BART5-5p) targets PUMA to promote survival of host cells (Choy et al., 2008). BART miRNAs also help establish EBV infection and transformation by modulating viral and host functions in B cells. Both miR-BART1-3p and miR-BART1-5p suppress adaptive immunity mediated by CD4^+^ T cells by targeting IL12B and LY75 (Skalsky et al., 2012; Vereide et al., 2014; Tagawa et al., 2016). CD8^+^ T cell responses are also modulated by miR-BART1-3p and miR-BART17-5p which target IFI30 and TAP2, respectively (Albanese et al., 2016). Thus, BART miRNAs enhance lymphomagenesis. Similarly, BART miRNAs potentiate tumorigenesis in epithelial cells (Cai et al., 2015; Kanda et al., 2015; Qiu et al., 2015; Yang et al., 2017). The notion that BART miRNAs positively regulate oncogenesis is generally accepted.

In DNA viruses such as herpesviruses, exosomes released from virus-infected cells contain virus-derived components that contribute to the growth of the virus itself and the establishment of viral infections by regulating the host’s immune system (Ansari et al., 2013). LMP1, which is an oncoprotein bound to cytoplasmic membranes, is incorporated into exosomes, and plays a role in cancer progression (Nanbo et al., 2013; Sato et al., 2017). EBV miRNAs are also released from infected cells *via* exosomes to regulate uninfected adjacent cells and promote their growth, which contributes to tumorigenesis (Pegtel et al., 2010; Higuchi et al., 2018; Nanbo et al., 2018; Nkosi et al., 2020).

### Deletion of BART miRNA in EBV-Related Lymphomas

It has been reported that the lack of certain genes in human T-cell leukemia virus type 1, human papillomavirus, and Merkel cell polyomavirus results in increased tumorigenicity (Narisawa-Saito and Kiyono, 2007; Moore and Chang, 2010; Kataoka et al., 2015). In contrast, only a few studies have reported specific gene deletions in EBV-associated malignancies (Alfieri and Joncas, 1987; Palser et al., 2015; Mine et al., 2017). However, in this next-generation sequencing era, whole viral genomes are easily sequenced directly from patient samples. We performed whole EBV sequencing by the hybrid capture method using 17,237 probes covering the entire EBV genome in EBV-infected peripheral blood and tumor tissues from patients with various EBV-associated diseases (Okuno et al., 2019; Mabuchi et al., 2021). Interestingly, 22 of 77 cases (35%) of CAEBV, which is a T- or NK-cell lymphoproliferative disease (Kimura et al., 2012; Kimura and Cohen, 2017; Quintanilla-Martinez et al., 2017), had a deletion of 73–49,847 bases in the EBV genome. A similar deletion was found in ENKTL (43%) and EBV-positive DLBCL (71%), which are lymphomas of NK- and B-cell origins, respectively. However, this intragenic deletion was not observed in infectious mononucleosis or post-transplant lymphoma, suggesting that this deletion is a common phenomenon in certain types of EBV-positive lymphoma. The rarity of the intragenic deletion has been reported in healthy individuals and infectious mononucleosis patients (Palser et al., 2015; Yajima et al., 2021).

In addition, EBV deletions were concentrated in the BART miRNA cluster regions (Figure 1). The most frequently deleted miRNAs were mir-BART6-5p and mir-BART6-3p, both of which negatively regulate the EBV immediate-early genes BZLF1 and BRLF1 (Iizasa et al., 2010). mir-BART18-5p and mir-BART20-5p also downregulate these immediate-early genes (Jung et al., 2014; Qiu and Thorley-Lawson, 2014). Mutated EBV lacking this region enhances lymphoma formation by inducing BZLF1 expression in xenograft models (Lin et al., 2015). Elevated BZLF1 due to loss of BART miRNAs may cause abortive lytic infection and promote lymphomagenesis (Munz, 2019).

In our genetic analysis, a group of lytic infection-related genes was also frequently missing outside the BART miRNA clusters, including core replication genes, which are essential for viral replication. We generated a mutant EBV strain lacking BALF5 (viral DNA polymerase catalytic subunit) (Narita et al., 2015) and used it to establish an LCL, which was then transplanted into immunodeficient mice (Okuno et al., 2019). The BALF5-deficient EBV produced lymphoma more frequently compared with the wild-type strain. Additionally, the BALF5-deficient LCL enhanced immediate-early/early gene expression, compared with the wild-type strain. These results suggest that deletion of BALF5, which is a core replication gene, induces the expression of lytic infection-related genes triggered by the immediate-early gene BZLF1 and promotes lymphoma formation. Multiple lytic infection genes, such as BNRF1, BGLF5, and BALF3, are involved in host genome instability (Manners et al., 2018; Xiong et al., 2020), and BHRF1 (viral BCL-2 homolog) and BCRF1 (viral interleukin-10 homolog) also promote cell proliferation (Xu et al., 2001; Zuo et al., 2011). EBV lacking core replication genes cannot produce virus particles or complete a lytic infection, but they can induce an abortive lytic infection and promote oncolytic infection by expressing a viral lytic infection gene (Munz, 2019; Murata et al., 2020). Thus, defective EBV strains may have some advantages during lymphomagenesis.

Interestingly, B95-8, which is the most potent laboratory strain, lacks most of the BART region (Figure 1; Baer et al., 1984; Klinke et al., 2014). Similar large deletions, including BART miRNA clusters, have been reported in patients with ENKTL and Hodgkin lymphoma (Peng et al., 2018; Kawatsuki et al., 2020). However, these defective viruses are relatively rarely associated with epithelial tumors (Cancer Genome Atlas Research Network (CGARN), 2014; Lin et al., 2014). The roles of BART miRNAs may differ between lymphoid and epithelial malignancies. The expression patterns of BART miRNAs depend on the infected cell lineage, and their expression levels vary widely among tumor types, with a 13-fold-increase in nasopharyngeal carcinoma and eightfold increase in gastric cancer relative to LCL and Burkitt lymphoma (Qiu et al., 2011).

Although intragenic deletions involving BART miRNAs have been detected in one-third of CAEBV patients, high expression levels of BART miRNAs are also seen in other patients with CAEBV (Kawano et al., 2013). Furthermore, Higuchi et al. (2018) reported that patients with EBV-positive DLBCL can be stratified according to the miR-BART13 expression level. Moreover, high expression of miR-BART13 was linked to shorter survival time (Higuchi et al., 2018). It is possible that BART miRNAs can contribute to disease progression by suppressing lytic EBV replication and host immune responses. They could also play a role in resistance to DLBCL therapies. On the other hand, loss of BART miRNA expression may also play a pivotal role in lymphoma formation during the early stages (Murata et al., 2020). In other words, cells harboring EBV miRNA deletions may have some advantages during lymphomagenesis as they induce an abortive infection leading to the immortalization of infected cells (Munz, 2019; Murata et al., 2020), at least in certain types of lymphomas. Once lymphoma develops, the loss of BART miRNA expression might not affect its progression and prognoses. Indeed, the overall survival time did not differ between CAEBV patients with and those without intragenic EBV deletions (Okuno et al., 2019).

## Conclusion

Epstein–Barr virus is a large DNA virus that encodes >80 genes and nearly 50 non-coding RNAs. Each non-coding RNA performs multiple and different functions, and their expression is regulated differentially according to cell type and micro-environment. EBV miRNAs have various functions and play pivotal roles in oncogenesis. It is clear that some viral miRNAs suppress lymphomagenesis, and that deletion of these miRNAs promotes lymphoma formation. Further research is necessary to elucidate the full roles of EBV miRNAs in tumorigenesis.

## Author Contributions

All authors contributed to the concept development process and to the writing and review of the manuscript and also gave final approval of the version to be published.

## Conflict of Interest

The authors declare that the research was conducted in the absence of any commercial or financial relationships that could be construed as a potential conflict of interest.
